# From Consultation to Shared Decision‐Making: Youth Engagement Strategies for Promoting School and Community Wellbeing

**DOI:** 10.1111/josh.12960

**Published:** 2020-11-12

**Authors:** Linda Sprague Martinez, Elizabeth Pufall Jones, Nico Connolly BA

**Affiliations:** ^1^ Macro Department Boston University School of Social Work, 264 Bay State Road Boston MA 02215; ^2^ Center For Promise, Wheelock College of Education and Human Development Boston University MA; ^3^ Strategic Initiatives and Partnerships Americas Promise Alliance, 1110 Vermont Avenue, N.W. Suite 900 Washington DC 20005

**Keywords:** youth engagement, youth voice, school health policy, school‐community partnerships, WSCC model

## Abstract

**BACKGROUND:**

The benefits of youth engagement are well documented. In this paper, we examine youth engagement in America's Promise Alliance's Every School Healthy initiative, a part of the Robert Wood Johnson Foundation's *Together for Healthy and Successful Schools Initiative (THSS).*

**METHODS:**

Six community acceleration sites were selected through a competitive grant‐making process. Sites were required to describe youth engagement strategies. A case study design was employed to examine how sites conceptualize youth engagement as well as youth engagement strategies employed across 6 sites. Data sources included observations, team member debriefs, and document review.

**RESULTS:**

There was variation in how *youth engagement* and *youth voice* are conceptualized in educational settings, and readiness for youth engagement. Sites actively solicited and implemented youth engagement resources and strategies.

**CONCLUSIONS:**

By failing to engage young people, well‐intentioned adults miss important opportunities. Youth engagement presents an exciting opportunity for school leaders, policymakers, and program planners to gain a deeper understanding of the factors that influence individual and community health and wellbeing and, in turn, helps them to develop responsive policies and programs.

Youth participation has been characterized in the literature as the process through which young people engage with and influence the organizations, institutions, and systems that impact their lives.^1^ The last decade has seen an increase in effort to increase youth participation in areas such as municipal governance,^2^, ^3^, ^4^ public health,^5^, ^6^, ^7^, ^8^ social work,^9^ child welfare,^10^ youth development,^11^, ^12^ and schools.^13^, ^14^, ^15^ Despite an interdisciplinary knowledge base describing the benefits of youth participation as well as the process of engaging youth and associated outcomes, young people still have few opportunities to meaningfully engage in decisions related to policies, programs, and services that impact their health and development.^16^, ^17^, ^18^ This is particularly true in the case of marginalized youth populations for whom existing community and school assets may be out of reach.^16^

In this paper, we explore the process of infusing youth participation in school‐community partnerships to accelerate school and community action to advance health through an illustrative case study of the America's Promise Alliance, *Every School Healthy (ESH)* initiative. ESH, by design, was intended to center young people employing the Whole School Whole Community Whole Child model (WSCC)^19^ and more specifically to promote authentic youth participation. Recognizing the value of young people's perspectives and wisdom, the initiative called for youth voice in decision‐making. This paper contributes to the school health literature by describing youth authentic participation as an important strategy for centering youth as part of the WSCC.^19^ How participation was conceptualized across local sites is explored. In addition, strategies partnerships can employ to both prepare for meaningful youth participation and assess their readiness for youth‐adult partnerships are presented.

A brief background on youth participation and voice is provided followed by an overview of the ESH initiative. The case study methodology employed is outlined and the results are presented and then discussed in the context of the extant literature. We conclude by discussing the limitations as well as the youth participation implications for school health.

Youth participation in schools has been defined as a partnership or collaboration between students and diverse adult stakeholders in the school setting.^13^ In the context of youth participation, young people, in this case students, are centered as assets who hold important knowledge and expertise needed to inform decision‐making.^20^, ^21^ The benefits of centering young people as leaders are many and have been documented for both youth and adults as well as organizations and institutions.^13^, ^18^ Youth develop sociopolitical awareness and increases in motivation, civic participation, professional development, skills, and competencies have been observed.^9^, ^13^, ^14^, ^22^ Adults meanwhile, see improvement in the quality of their relationships with youth as well as their understanding of youth.^13^ Organizations and institutions yield relevant policies and programs and improvements in organizational culture have been seen.^13^

Given the benefits of the youth participation and its alignment with the mission and values of America's Promise Alliance (APA), the organization sought to infuse *youth voice* in the ESH initiative in addition to community stakeholder engagement. Youth participation has been referred in the education literature as student or youth voice. Like youth participation and engagement, it is not consistently defined in practice. For example, it is not merely the collection of youth priorities or opinions by adults. More so, it refers to meaningful engagement in decision‐making and goal‐setting that leads to changes in school programs and policies that reflect youth priorities.^23^ Moreover, like other participatory approaches, initiatives to harness youth voice are rooted in critical pedagogy, and recognize the need for the redistribution of power in school settings, whereby students are driving change and decision‐making in partnership with supportive, caring adults.^23^

In 2017, with funding from the Robert Wood Johnson Foundation, APA launched ESH. ESH is a multi‐year, multisector initiative that was designed to catalyze a national movement advancing school health. The initiative included 3 primary objectives and was grounded in the notion that all schools can be healthy schools. The first objective was focused on local action to advance health equity. The team recognized solutions to pronounced inequities in both health and education demand investment in local knowledge and decision‐making. As such, 6 unique school‐community partnerships from across the country were selected through a competitive application process. The second objective was to promote bidirectional capacity building and cross‐site learning, whereby practitioners from local sites as well as youth and partners, researchers, and school leaders were learning from one another—both informing and adopting best practices. The learning laboratory, as it was developed, was then extended across sites to form a multisite learning community. Finally, broad dissemination was employed to further contextualize and amplify learnings. Real‐time sharing occurred with APA partners across the nation through webinar and local dialogues as well as, traditional earned media, listservs, and social media. At the center of this initiative were young people, students. As part of the application, process sites were asked to describe their plans for ensuring youth voice in their efforts. The level of youth engagement at each site was assessed. Using instrumental case study design, we describe the ranking process, and explore how youth engagement was conceptualized across sites. Youth engagement strategies and perceived technical assistance needs related to youth engagement are also examined. What emerged was a story about the promises and challenges associated with youth engagement as well as the need to explore organizational readiness for youth engagement.

## METHODS

Instrumental case study design was employed to qualitatively examine how ESH sites conceptualize youth engagement as well as youth engagement strategies employed across 6 sites.^24^, ^25^ This social science research is designed for the study of a case in a real‐life, present‐day setting.^24^, ^25^ Data sources included meeting and site observations; team member debriefs as well as document review (Table 1). Procedures associated with each source of data are described in detail below. This study was determined to be non‐human subjects research data collected through debriefs were focused on program processes and were not designed to be generalizable but more so to inform our work with the sites.

**Table 1:** Data Sources

Data Sources	
Project documents	Project planning documents, meeting agendas, meeting notes, applications, project reports, site reports, newsletters, and media
Observations	APA project meetings and site meetings
APA Staff and Site debriefs	Debriefs with APA staff and acceleration site staff

### Procedures

The overall goal of observations was to explore youth engagement strategies as well as conceptualization of youth engagement and perceived technical assistance needs. Observations were planned and conducted by the youth engagement specialist at in‐person site visits, in addition, conference calls and remote meetings were observed. Comprehensive notes were taken during and at the culmination of each observation. Observation notes were typed and then analyzed thematically.

Similar to the observations, document review was designed to explore how sites conceptualized youth engagement as well as strategies and technical assistant requests. Project documents included: ESH program descriptions developed by APA, applications and applicant narratives, evaluation reports, and learning agenda reports and grant reports from sites as well as materials released via websites, newsletters, and social media.

Finally, themes were captured from debriefs with APA staff over the course of the project. In addition, debriefs with sites about their youth engagement technical assistance needs were conducted. The data collection and analysis, were continuous and emergent themes were shared with APA staff over the course of the initiative. Similarly, site‐specific themes were shared with sites after meetings and observations.

### Data Analysis

Two types of data analysis were employed, holistic analysis of the whole case and embedded analysis of specific parts of the case.^24^ A chronology of events was examined in addition to key themes to assess the complexity of the case.^24^ Each data source was coded inductively, focusing on the data itself without preconceived categories.^26^, ^27^ Documents as well as notes from direct observation and debriefs were reviewed and analyzed thematically.

Each document was read multiple times to search for meaningful patterns,^26^ and to reflect on questions and reactions to the codes.^28^ Themes were discussed with APA staff over the course of the project to ensure consistency in the analytic approach.^29^ Codes were developed by labeling and naming selected text and then sorted into possible themes. Relationships were explored between themes across levels and refined to ensure the data within each was cohesive and distinct.^26^, ^27^ The larger story within the data was then identified and illustrative examples of engagement, as well as text, was selected to produce a coherent story within and across the identified themes from each data source.^26^

## RESULTS

How youth engagement was assessed across the site application process is presented accompanied by youth engagement levels. We then highlight the approach to technical assistance strategies used by APA and its core partner organizations as well as youth engagement activities employed across the selected sites. The section concludes with an overview of the challenges of youth engagement as described by sites.

**Figure 1:**
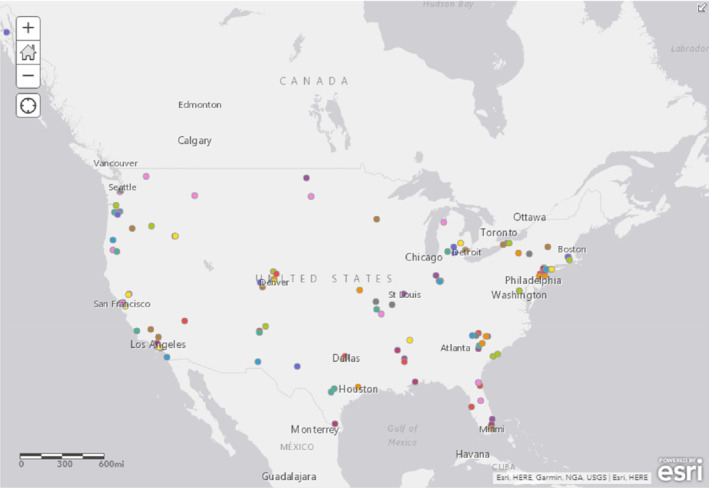
Applicant Map

Six acceleration sites were selected from a total of 138 eligible applications from 20 states. Figure 1 illustrates the geographic spread of the applicants. The application responses were coded thematically to explore themes across applicant sites. The most commonly mentioned health and educational challenges cited by the 138 applicants included: poverty; race, racism and racialization; transportation; food insecurity; trauma; obesity; health care access; mental health; and low graduation rates. Applications were scored by a group of reviewers including youth, APA staff, and core partners. Each application was scored by 4 individuals.

### Assessing Youth Engagement

Youth engagement was scored separately from the overall application and did not impact the overall scoring. According to project documents, opportunities for youth involvement at both the national and local levels were intentionally built in. The goal was to work with sites to build their capacity for youth engagement throughout the process:

*…the campaign [initiative] will meet communities where they are when it comes to youth engagement. Some communities are ready for and have a need for infusing youth voice into their discussions, others want and need to have youth at their decision‐making table, and still others want to engage youth in pursuing inquiries that are needed for making decisions*. [APA document]

The hope was that focusing on meaningful youth engagement would:

*Catalyze shifts in school, district, and broader community practices and policies that incorporate more youth voice in decision‐making processes and support youth as agents of change for healthy environments…* [APA document]

*Increased knowledge, attitudes, and beliefs of direct stakeholders (eg, youth, parents, school leadership) of the factors that contribute to a healthy school environment and the key levers for systems‐level change*. [APA document]

According to project notes, the level of youth engagement was ranked for each application using content analysis and scoring. The content was coded based on the nature of the activities using the levels described in Table 1. These activities were informed by the literature on youth engagement, community research engagement, and community readiness.^30^, ^31^, ^32^

The review process involved scanning application content to determine the presence of youth engagement activities described in the criteria outlined in Table 2. Once the numeric value of level associated with each content code was applied, average scores were generated for each application. Final scores were rounded, ranging from 1 to 3. The mean score among sites was 1.9; 38% of sites scored 1; 34% score 2 and 28% scored 3. Of the sites scoring 1, the majority of conceptualized youth voice as surveying youth or exploring youth thinking. However, in these cases, youth were not engaged in decision‐making. Adults were interpreting survey results, interviews, and focus group findings as a strategy to harness youth voice.

**Table 2:** Levels of Youth Engagement

Level	Description [APA Presentation]
1	Youth are not at the table. Community meetings are planned to inform them of proposed activities. Data is collected from them and analyzed by the action group. Leaders from youth‐serving organizations are included to speak on behalf of young people.
2	Strategies have been developed to inform youth of activities and to solicit their feedback. Youth have been identified to be part of the process. This might include a youth board. The process to engage young people is not clear. Youth engaged in past and present efforts have been the “usual suspects”, perhaps the group “skims off the top”. There is no training or capacity building in place for youth, they are decorative and not necessarily involved in the decision‐making process.
3	Groups have procedures in place for recruiting diverse segments of the youth population. Youth are compensated for their role. They are part of the decision‐making process. There is team‐building training in place for youth and adults designed to enhance communication, coordination, and trust relationships and reduce adultism. Youth are engaged across all aspects of the work, which reflects their priorities.

Of the 138 sites, 16 finalists were selected. Youth engagement scores varied among the 16 sites. The mean score was 1.6. Half of the 16 (50%), scored 1, 37.5% scored 2, and 12.5% scored 3. The APA team and core partners met to narrow the 16 applications to 6. All applicants were discussed and finalists were selected using a consensus decision‐making process with the group, that involved dot polling followed by group discussion. The process continued until the pool was narrowed to 6.

The 6 finalists participated in telephone interviews with APA staff. Sites were asked a number of clarification questions associated with their youth engagement plans in an effort to further hone their level. For example, one site was asked:

- *How will youth be engaged in the decision‐making process?*
- *Will [youth] teams only provide feedback or will they be encouraged to lead the way?*
- *How will you ensure those [youth] most impacted will be at the [decision‐making] table?* [review documents]

Interview responses were reviewed and coded using the process previously described. Scores were then adjusted. The 6 selected sites participated in telephone meetings with technical assistance (TA) providers including the youth engagement specialist.

### Youth Engagement Technical Assistance

Technical assistance (TA) began with an introduction to youth engagement. Hart's ladder^30^ was used to illustrate the spectrum of youth engagement. Youth engagement TA by level can be seen in Table 2. The training was intended to create a shared understanding of youth engagement across sites. As noted in project documents:

*There are many terms that get used interchangeably when referring to how young people are involved with a program—eg, Youth Engagement, Youth Participation, Youth Voice, Youth Leadership, Youth Empowerment*. [project report]

Planning notes indicate that after the initial training individualized plans were developed based on level goals and TA activities outlined in Table 2. For those at level 3, the youth engagement specialist served as a thought partner to help site staff develop strategies to maintain and perhaps expand the site's reach in their communities. For level 2 sites, the goal was to advance their existing work (Table 3).

**Table 3:** Technical Assistance Goals and Activities

Level & #of sites	Technical Assistance Goals and Activities [APA presentation]
1 (N = 2)	Goal: Create awareness: Sites will be able to define youth voice and to describe the benefits of youth participation in decision‐making and action efforts for their stakeholders. Sites will begin to develop strategic plans for engagement leveraging existing partner assets and community resources. Sites will understand what adultism is and how it operates. Activities: Direct consultation, resource sharing, webinars and trainings.
2 (N = 2)	Goal: Increase site readiness and capacity for harnessing youth voice. Implement strategies for engaging youth in decision‐making and action efforts. Sites will employ strategies for addressing adultism. Activities: Direct consultation, resource sharing, curriculum development, development of formalized strategies for recruitment youth and adults, and training procedures. Provision of teambuilding, communication, and relationship development strategies.
3 (N = 2)	Goal: Formalize, evaluate, and disseminate best practices and strategies for youth‐adult partnerships and engaging youth in decision‐making and action efforts. Supporting the development of structure to expand and formalize the work. Activities: Direct consultation, resource sharing, advising on strategic planning, participatory Evaluation, and dissemination as well as sustaining best practices.

*Some acceleration sites have strong programming in other areas (eg, parent engagement). In this instance the youth engagement consultant [specialist] offers them support to modify the existing materials and curriculum so that it is appropriate for young people and the goals associated therewith*. [internal initiative report]

*…provided a 2‐day youth assessment training to young people from 2 high schools as well as a train‐the‐trainer workshop for site staff to provide these trainings in the future*. [external initiative report]

*…[TA materials] not only served as a resource but also provided a template for the “State of Child Report” that the … site was working on, wherein the young people talk about the biggest issues that impact young people in the city and the changes needed to solve those issues*. [external initiative report]

Level one sites, the goal was to provide a steady flow of information and to begin to explore strategies to plan.

*…[TA] provided them with strategies, concepts, and framework that they had not thought through before. For example, one of the sites mentioned that they had not thought about how youth who are more vulnerable backgrounds (eg, from low‐income families, are Black/Hispanic, have faced more ACEs) take more risk when they engage and make their voices heard as compared to youth who are less vulnerable. This led some sites to more careful planning in the first year* [external initiative report]

*Another project director mentioned that the idea that the site could involve youth equipping them with basic research skills and data collection methodology so they can analyze and interpret their own data was new to the project director*. [external initiative report]

Overall, data indicate sites benefited from youth engagement frameworks and examples as well as brainstorming trouble shooting and direct training.

*…Interviewees said that engaging youth as leaders has the potential to bring new insights to the health and well‐being of young people, provide a broader understanding of young people's wellness, and increase their skills, confidence, and agency. Most importantly, engaging them made sure “that interventions and programs in school settings are actually addressing the needs of young people as they state them” (site project director)*. [external initiative report]

### Site Youth Engagement Strategies

All 6 acceleration sites have implemented or are in the process of implementing youth leadership collectives.

*Youth‐led Councils have been formed in 2 high schools and one middle school… All 3 student wellness councils have been trained in health equity and have chosen their top health issues to focus on next school year…* [site report]

Youth groups are referred to as action groups, ambassadors, councils, and advisors. The structure and function of youth leadership collectives vary across sites. Activities, for example, range from service and peer education to research and policy advocacy. Youth engagement activities are shown in Table 4 illustrate examples of the role youth are playing across each of the 6 sites.

**Table 4:** Youth Engagement Activities

Activities	Illustrative quotes [site reports]
Policy advocacy	*Recently, students emailed and sent postcards to their representatives in support of HB 2015 (Driver's Licenses for All) and explained why voting YES on this bill is important for the well‐being of the Latino community*. *We work with youth advisory/leadership groups at specific schools and have developed a youth advisory group that helps to direct our work. We are currently working with our advisory group to develop a report card assessment of how [city] responds to the needs of young people*. *Our high school students are now guardians of wellness policy and the fact that …High School Change Agents' initiative went district‐wide in just 1 year is a huge highlight*.
Research	*Our advisory group is developing a survey tool and developing strategies to encourage their peers across the city to take the survey*. *…we plan to convene youth during the school day to work with and interpret the results of our resilience data alongside adults. [site] will facilitate this effort, and we believe it will lead to powerful discovery and understanding about ways to increase resilience among our students and staff in schools*.
Education	*Our advisory group is also helping to develop a social media strategy to get young people to engage in discussions about our community's strengths and barriers to youth well‐being in [city]*. *Students have begun planning a week‐long summer camp aimed at developing the leadership skills of their peers*. *[Acceleration site partner] has been successful in beginning to create a strong youth advisory council that will be a conduit to youth voice specifically around mental health concerns and stigma*. *[Site] was able to successfully launch the first cohort, bringing together 28 students from 7 different schools throughout the region, including an alternative high school. These students went through 4 days of intensive community building, facilitation, story sharing, identity development, and action planning. These students will now be leaders in the … Youth Ambassador program, and assist in training future cohorts*.
Service	*… students have been collecting donation items to create care packages for unhoused individuals in [name of*] *County. This service project was adopted by the group because students have witnessed on personal accounts the impact of poverty in the community. Students promoted the project in their schools to collect donations from their peers and teachers*.

### The Challenges of Youth Engagement

Youth engagement was not without its challenges. Sites described a number of impediments to establishing partnerships with young people and engaging them in meaningful ways. These challenges varied but in most cases were structural. Namely, community‐level factors such as transportation and organizational factors related to interagency collaboration, resource scale‐up, and staffing issues. Individual factors related to behavior, capacity, and life circumstances were also noted. Table 5 provides a snapshot of challenges by theme.

**Table 5:** Challenges

Challenges	Illustrative quotes [acceleration site reports]
Community factors	*…[city name] is physically a large city and has poor transportation infrastructure. Most young people we work with do not have regular access to transportation making it difficult for them to commit to regular meetings*. *Our community lacks a comprehensive strategy or framework to advance the needs of young people. There continues to be several different community organizations with a youth focus, many of these organizations address the same topics. There are often community meetings, events or panels addressing the same issues that occur within a short amount of time. As a result, much of the progress is not shared between organizations or throughout our community as a whole. Additionally, many organizations seem to be competing for the same (or similar) funding opportunities, making it difficult to promote and sustain a collaborative approach*.
Organizational factors	*We serve youth in 3 different school districts, and it is difficult to find central locations. Staff capacity does not allow for us to have more than 1–2 leadership meetings per month and they must rotate to different central locations to give youth a higher chance to participate*. *Navigating appropriate scaling of the YAA was a challenge*. *Another challenge was that a few School Wellness Coordinators/Mountain Movers did not necessarily recruit all students that we wanted to target‐ the disengaged students*.
Individual factors (student and staff)	*The circumstances of their [youth] lives and the challenges that they face may make it difficult for many of them to maintain interest in this work and many who are interested may not have the family support to regularly engage in the work*. *Challenges in engaging youth mostly revolved around timing. Starting in the spring of the school year is not ideal. Students are burned out and busy with state testing, advanced placement tests, and end‐of year activities. School staff is also burned out and trying to squeeze in any outstanding tasks before the end of the school year*. *Another challenge was behavioral issues among the middle school students and a chaotic school environment, which made it difficult to get through workshops. During these meetings, [acceleration site] staff observed greater support would be required for classroom management*.

## DISCUSSION

Consistent with the literature, we found there is a great deal of variation in how youth voice and youth engagement are conceptualized.^23^ Youth engagement spanned the spectrum of Hart's Ladder^30^; however, this was not always black and white. Engagement levels varied within programs and across activities. Even in cases where sites had *youth‐initiated* activities and programs, there were instances when youth engagement was characterized as *tokenism* or *assigned and informed*.^30^ This was likely associated with having youth engagement champions in settings with little overall infrastructure for youth engagement. This points to the importance of organizational readiness for youth engagement.

There are multiple elements that comprise an organization. For example, McKinley lays out 7 organizational elements: *structure, systems, strategy, skill, staffing, style, and shared values*.^33^ As such, even organizations with youth engagement embedded in their values, may not have the structures or skills to support authentic youth engagement.^33^ External factors to the organization, such as transportation and interorganizational collaborations also served as barriers to readiness for youth engagement. Systematically assessing readiness for youth engagement across organizational elements, as well as planning tools such as Strengths Weaknesses Opportunities and Threats (SWOT) analysis may be important strategies for identifying both external and internal factors that may influence engagement.

Overall, sites found Hart's Ladder to be a framework by which to understand engagement and provided clear goals to target. Some sites used the graphic of the Ladder with youth to reflect on where they were with respect to programming. Exploring the rungs of the ladder together, youth and adults evaluated their engagement and were able to collaboratively generate partnership goals as well as strategies for arriving at their desired destination. Similarly, sites benefited from resources and TA activities aimed at examining how adultism operates within systems. Authentic youth engagement calls for adults ready to tackle ideology, systems and structures that proliferate adultism and impede engagement. At the end of the day, this work takes both time and intentionality. Organization readiness assessments may facilitate efforts to engage youth authentically.

### Limitations

This work is not without limitation. We present an analysis of observational data and program documents coded by a single reviewer, which may have introduced bias. To compensate for this limitation study, findings have been reviewed by and discussed with APA staff to explore the extent to which finding reflect their lived experience. In addition, the triangulation of multiple data sources has helped to ensure validation.^34^ Despite limitations, future studies exploring changes in youth engagement over time through both observational and interview data may provide important insight. Similarly, implementation studies may help to discern practices and strategies most effective in facilitating meaningful youth engagement.

### Conclusions

As we strive to implement WSCC strategies for improving outcomes,^19^ failing to meaningfully engage young people we, well‐intentioned adults, miss important opportunities. Youth engagement presents an exciting prospect for school leaders, policymakers, and program planners to gain a deeper understanding of the factors that influence individual and community health and wellbeing and, in turn, helps them to develop responsive policies and programs. It also helps to cultivate the next generation of leaders. However, getting there requires intentionality and incremental systems change.

### IMPLICATIONS FOR SCHOOL HEALTH

Consistent with the literature, we found efforts to create healthy environments for students that fail to meaningfully engage them to run the risk of employing adult‐centric approaches.^35^ All sites noted, engaging those who are most marginalized in the decision‐making process can help to ensure the relevance of practices and policies to promote health for everyone. Youth engagement presents a promising strategy for school health education and health promotion efforts. Moreover, youth engagement is associated with positive health and developmental outcomes for youth.^18^

In the context of COVID‐19, we, adults, need to be particularly mindful of what it means to authentically engage young people. The goal of any social system is to maintain equilibrium. Youth engagement is complicated because it challenges norms within social systems, including schools. Today, systems are in crisis and having to adapt to a new landscape. As such, we run the risk of setbacks if we do not continue to both think and act intentionally about how we collaborate, partner, and engage with youth.

### Human Subjects Approval Statement

This research was determined not to be human subjects research by the Boston University Charles River Campus Institutional Review Board.

### Conflict of Interest

Linda Sprague Martinez is a research consultant for Americas Promise Alliance and the Community Clinic, Inc. She was previously a research consultant for RAND and Human Impact Partners.

## References

[1] Checkoway BN . What is youth participation? Child Youth Serv Rev. 2011;33(2):340‐345.

[2] Augsberger A , Collins ME , Gecker W . Best practices for youth engagement in municipal government. Natl Civ Rev. 2017;106(1):9‐16.

[3] Augsberger A , Collins ME , Gecker W . Engaging youth in municipal government: moving toward a youth‐centric practice. J Community Pract. 2018;26(1):41‐62.

[4] Collins ME , Augsberger A , Gecker W . Youth councils in municipal government: examination of activities, impact and barriers. Child Youth Serv Rev. 2016;65:140‐147.

[5] Sprague Martinez L , Tang Yan C , McClay C , Varga S , Zaff JF . Adult reflection on engaging youth of color in research and action: a case study from five US cities. J Adolesc Res. 2020 10.1177/0743558420906086 [E‐pub ahead of print].

[6] Sprague Martinez LS , Gute DM , John Ndulue U , Seller SL , Brugge D , Peréa FC . All public health is local revisiting the importance of local sanitation through the eyes of youth. Am J Public Health. 2012;102(6):1058‐1060. 22515855 10.2105/AJPH.2011.300635 PMC3411102

[7] Sprague Martinez LS , Reich AJ , Flores C , et al. Critical discourse, applied inquiry and public health action with urban middle school students: lessons learned engaging youth in critical service‐learning. J Community Pract. 2017;25(1):68‐89. 10.1080/10705422.2016.1269251 PMC10168677 37168989

[8] Tang Yan C , Moore de Peralta A , Bowers EP , Sprague Martinez L . Realmente tenemos la capacidad: engaging youth to explore health in The Dominican Republic through photovoice. J Community Engagem Scholarsh. 2019;12(1):8.

[9] Richards‐Schuster K , Pritzker S . Strengthening youth participation in civic engagement: applying the convention on the rights of the child to social work practice. Child Youth Serv Rev. 2015;57:90‐97.

[10] Augsberger A , Toraif N , Springwater JS , Koshinsky GH , Martinez LS . Strategies for engaging youth currently and formerly in foster cae in child welfare policy advocacy: lessons from the New England youth coalition (NEYC). Child Welf. 2019;97(6):251‐270.

[11] Ozer EJ . Youth‐led participatory action research: developmental and equity perspectives. Adv Child Dev Behav. 2016;50:189‐207. 26956074 10.1016/bs.acdb.2015.11.006

[12] Ozer EJ . Youth‐led participatory action research: overview and potential for enhancing adolescent development. Child Dev Perspect. 2017;11(3):173‐177.

[13] Griebler U , Rojatz D , Simovska V , Forster R . Effects of student participation in school health promotion: a systematic review. Health Promot Int. 2017;32(2):195‐206. 24395957 10.1093/heapro/dat090

[14] Kornbluh M , Ozer EJ , Allen CD , Kirshner B . Youth participatory action research as an approach to sociopolitical development and the new academic standards: considerations for educators. Urban Rev. 2015;47(5):868‐892.

[15] Mandel LA , Qazilbash J . Youth voices as change agents: moving beyond the medical model in school‐based health center practice. J Sch Health. 2005;75(7):239‐242. 16102085 10.1111/j.1746-1561.2005.00031.x

[16] Checkoway B , Richards‐Schuster K . Youth participation for educational reform in low‐income communities of color. 2006 Available at: http://citeseerx.ist.psu.edu/viewdoc/download;jsessionid=C5A079AC4F849CD1CFBFE2A094FE2F8D?doi=10.1.1.556.6477&rep=rep1&type=pdf. Accessed September 8, 2020.

[17] Ozer EJ , Newlan S , Douglas L , Hubbard E . “Bounded” empowerment: analyzing tensions in the practice of youth‐led participatory research in urban public schools. Am J Community Psychol. 2013;52(1–2):13‐26. 23444005 10.1007/s10464-013-9573-7

[18] Sprague Martinez L , Richards‐Schuster K , Teixeira S , Augsberger A . The power of prevention and youth voice: a strategy for social work to ensure youths' healthy development. Soc Work. 2018;63(2):135‐143. 29373731 10.1093/sw/swx059

[19] Lewallen TC , Hunt H , Potts‐Datema W , Zaza S , Giles W . The whole school, whole community, whole child model: a new approach for improving educational attainment and healthy development for students. J Sch Health. 2015;85(11):729‐739. 26440815 10.1111/josh.12310 PMC4606766

[20] Checkoway B , Richards‐Schuster K , Abdullah S , et al. Young people as competent citizens. Community Dev J. 2003;38(4):298‐309.

[21] Finn JL , Checkoway B . Young people as competent community builders: a challenge to social work. Soc Work. 1998;43(4):335‐345.

[22] Chan B , Carlson M , Trickett B , Earls F . Youth participation: a critical element of research on child well‐being In: Lerner RM, Benson PL, eds. Developmental Assets and Asset‐Building Communities. New York, NY: Springer; 2003:65‐96.

[23] Conner JO , Ebby‐Rosin R , Brown AS . Introduction to student voice in American education policy (reprinted from NSSE yearbook, vol 114, pgs 1‐18, 2015). Teach Coll Rec. 2015;117(13):1‐18.

[24] Creswell JW . Qualitative Inquiry and Research Design: Choosing among Five Approaches. Thousand Oaks, CA: Sage Publications; 2013.

[25] Crowe S , Cresswell K , Robertson A , Huby G , Avery A , Sheikh A . The case study approach. BMC Med Res Methodol. 2011;11(1):1‐9. 21707982 10.1186/1471-2288-11-100 PMC3141799

[26] Braun V , Clarke V . Using thematic analysis in psychology. Qual Res Psychol. 2006;3(2):77‐101.

[27] Patton MQ . Qualitative Evaluation and Research Methods. 2nd ed. Newbury Park, CA: Sage Publications; 1990.

[28] Charmaz K . Constructing Grounded Theory. Thousand Oaks, CA: Sage Publications; 2006.

[29] Boyatzis RE . Transforming Qualitative Information: Thematic Analysis and Code Development. Thousand Oaks, CA: Sage Publications; 1998.

[30] Hart RA . Stepping back from ‘the ladder’: reflections on a model of participatory work with children In: Reid A, Jensen BB, Nikel J, Simovska V, eds. Participation and Learning. New York, NY: Springer; 2008:19‐31.

[31] Shier H . Pathways to participation: openings, opportunities and obligations. Child Soc. 2001;15(2):107‐117.

[32] Sprague Martinez LS , Freeman E , Perea FC . From engagement to action: assessing community readiness for disparities mobilization. J Health Dispar Res Pract. 2012;5(2):9.

[33] McKinsey & Company . Enduring Ideas: The 7‐s Framework. New York, NY: McKinsey & Company; 2008 Available at: https://www.mckinsey.com/business‐functions/strategy‐and‐corporate‐finance/our‐insights/enduring‐ideas‐the‐7‐s‐framework. Accessed September 8, 2020.

[34] Leech NL , Onwuegbuzie A . An array of qualitative data analysis tools: a call for data analysis triangulation. Sch Psychol Q. 2007;22(4):557‐584.

[35] Castrechini S , Gardner JW , Ardoin NM . Youth resource mapping: partnering with service providers and youth to understand the supply and demand for youth services in a local context. Penn GSE Perspect Urban Educ. 2011;8(2):3‐10.

